# Pomalidomide, dexamethasone, and daratumumab in relapsed refractory multiple myeloma after lenalidomide treatment

**DOI:** 10.1038/s41375-020-0813-1

**Published:** 2020-05-06

**Authors:** David S. Siegel, Gary J. Schiller, Christy Samaras, Michael Sebag, Jesus Berdeja, Siddhartha Ganguly, Jeffrey Matous, Kevin Song, Christopher S. Seet, Giampaolo Talamo, Mirelis Acosta-Rivera, Michael Bar, Donald Quick, Bertrand Anz, Gustavo Fonseca, Donna Reece, William E. Pierceall, Weiyuan Chung, Faiza Zafar, Amit Agarwal, Nizar J. Bahlis

**Affiliations:** 1 John Theurer Cancer Center, Hackensack University Medical Center, Hackensack, NJ USA; 2 David Geffen School of Medicine at University of California, Los Angeles, CA USA; 3 Cleveland Clinic, Cleveland, OH USA; 4 McGill University Health Centre, Montreal, QC Canada; 5 Sarah Cannon Research Institute, Nashville, TN USA; 6 The University of Kansas Cancer Center, Fairway, KS USA; 7 Colorado Blood Cancer Institute, Denver, CO USA; 8 Vancouver General Hospital, Vancouver, BC Canada; 9 Penn State Hershey Cancer Institute, Hershey, PA USA; 10 Fundación de Investigación, San Juan, PR USA; 11 Stamford Hospital, Stamford, CT USA; 12 Joe Arrington Cancer Research and Treatment Center, Lubbock, TX USA; 13 Tennessee Oncology, Chattanooga, TN USA; 14 Florida Cancer Specialists, St. Petersburg, FL USA; 15 Princess Margaret Cancer Centre, Toronto, ON Canada; 16 Bristol-Myers Squibb, Summit, NJ USA; 17 Arnie Charbonneau Cancer Research Institute, University of Calgary, Calgary, AB Canada

**Keywords:** Myeloma, Phase II trials

## Abstract

Patients with multiple myeloma who have relapsed after or become refractory to lenalidomide in early treatment lines represent a clinically important population in need of effective therapies. The safety and efficacy of pomalidomide, low-dose dexamethasone, and daratumumab was evaluated in lenalidomide-pretreated patients with relapsed or refractory multiple myeloma (RRMM) after one to two prior treatment lines in the phase 2 MM-014 study. Patients received pomalidomide 4 mg daily from days 1–21 and dexamethasone 40 mg weekly (28-day cycles). Daratumumab 16 mg/kg was administered per label. Primary endpoint was overall response rate (ORR); secondary endpoints included progression-free survival (PFS) and safety. Per protocol, all patients (*N* = 112) had received lenalidomide in their most recent prior regimen (75.0% lenalidomide refractory). ORR was 77.7% (76.2% in lenalidomide-refractory patients); median follow-up was 17.2 months. Median PFS was not reached (1-year PFS rate 75.1%). The most common hematologic grade 3/4 treatment-emergent adverse event was neutropenia (62.5%). Grade 3/4 infections were reported in 31.3% of patients, including 13.4% with grade 3/4 pneumonia. These results demonstrate the safety and efficacy of pomalidomide-based therapy as early as second line in patients with RRMM, even immediately after lenalidomide failure, indicating that switching from the immunomodulatory agent class is not necessary.

## Introduction

Advances in therapy have led to improved survival in multiple myeloma (MM); however, nearly every patient will relapse following initial treatment [1–3]. Although most patients will have received frontline treatment with lenalidomide-based regimens, patients with lenalidomide-refractory disease have been poorly represented in recent phase 3 relapsed or refractory MM (RRMM) clinical trials [1, 4–9]. Management of these patients remains challenging due to the availability of multiple treatment options combined with factors such as disease aggressiveness, patient age, and response to previous antimyeloma therapies [3, 10]. In addition, effective treatment of early RRMM is critical because patient outcomes worsen with each relapse, and the interval between relapses shortens with each subsequent line of treatment [2, 3, 11]. Therefore, patients who have become refractory to lenalidomide in early treatment lines are a clinically relevant population in need of proven and effective therapies [1, 4–9].

Pomalidomide, an immunomodulatory agent, exerts potent, direct tumoricidal, and immune-stimulating effects through binding to its molecular target cereblon, a component of the CRL4 E3 ubiquitin ligase, and subsequent degradation of the transcription factors Ikaros and Aiolos [12, 13]. The antitumor and immune-stimulating properties of pomalidomide are distinct from those of lenalidomide; pomalidomide has different substrate degradation kinetics, increased binding affinity to cereblon, and a different gene modulation profile [12–15]. Pomalidomide also has activity in lenalidomide-resistant myeloma cell lines and animal models and has demonstrated efficacy in patients refractory to lenalidomide in clinical trials [13, 14, 16–20].

Pomalidomide was initially approved in combination with dexamethasone for the treatment of patients with RRMM and ≥2 prior therapies (including lenalidomide and a proteasome inhibitor in the United States and lenalidomide and bortezomib in the European Union) [21, 22]. More recently, various pomalidomide-based triplet regimens have received regulatory approval. The combination of pomalidomide, dexamethasone, and daratumumab is approved in the United States for the same indication as the doublet regimen, as is the combination of pomalidomide, dexamethasone, and elotuzumab [23, 24]. Findings from the recent phase 3 OPTIMISMM trial that demonstrated a significantly prolonged progression-free survival (PFS) with pomalidomide, bortezomib, and dexamethasone (PVd) vs Vd (11.2 vs 7.1 months; HR, 0.61 [95% CI, 0.49–0.77]; *p* < 0.001) in patients with one to three prior regimens recently led to approval of PVd in several jurisdictions, including in the European Union and Japan, for the treatment of patients with RRMM who received ≥1 prior regimen [21, 25, 26].

Daratumumab, an anti-CD38 monoclonal antibody, exerts direct on-tumor and immunomodulatory activity [27–30]. Daratumumab was approved in the United States for the treatment of RRMM in combination with pomalidomide and dexamethasone based on results of the phase 1b EQUULEUS/MMY1001 RRMM trial [23, 31]. In this study of patients with heavily pretreated RRMM (median, four prior lines of therapy), the triplet combination led to an overall response rate (ORR) of 60%, including an ORR of 58% in patients who were refractory to both a proteasome inhibitor (PI) and an immunomodulatory agent [31]. Median PFS was 8.8 months, and median overall survival (OS) was 17.5 months. However, this regimen has not been extensively studied in earlier lines of therapy or in patients who became refractory to lenalidomide-based therapy immediately prior to study entry.

The phase 2 MM-014 trial investigated the outcomes of sequencing pomalidomide-based therapy immediately after lenalidomide failure in early treatment lines. Here we report safety and efficacy findings from cohort B, in which patients relapsed from or refractory to lenalidomide in their first or second treatment line received pomalidomide, low-dose dexamethasone, and daratumumab.

## Methods

### Study design and patients

MM-014 is a phase 2, nonrandomized, multicenter, open-label clinical trial with three cohorts conducted at 49 study sites in the United States, Canada, and Japan. Patients in cohort A received pomalidomide plus low-dose dexamethasone. Patients in cohort B received pomalidomide, low-dose dexamethasone, and daratumumab. Cohort C (currently enrolling) is a Japanese-only arm of the study; patients will receive pomalidomide, low-dose dexamethasone, and daratumumab. Patients were not allocated across cohorts; rather, cohort B was added to the trial via protocol amendment after the full accrual of cohort A, and cohort C was added after the full accrual of cohort B.

Patients eligible for inclusion in cohort B of MM-014 were ≥18 years of age with documented MM diagnosis, measurable disease (serum M-protein ≥0.5 g/dl or urine M-protein ≥200 mg/24 h), and Eastern Cooperative Oncology Group performance status ≤2. In addition, patients were required to have had one or two prior lines of antimyeloma therapy, documented progressive disease (PD) during or after their last line of therapy, and treatment with a lenalidomide-containing regimen for ≥2 consecutive cycles as their most recent regimen. Patients who relapsed after or were refractory to lenalidomide were eligible for inclusion. Refractory disease was defined as nonresponsive to therapy or as PD within 60 days of the last dose.

Key exclusion criteria included prior treatment with pomalidomide or daratumumab or hypersensitivity to thalidomide, lenalidomide, dexamethasone, or monoclonal antibodies. The following laboratory abnormalities were exclusionary: absolute neutrophil count <1 × 10^9^/l, platelet count <75 × 10^9^/l (<30 × 10^9^/l for patients in whom ≥50% of bone marrow nucleated cells were plasma cells), corrected serum calcium >2.875 mmol/l (11.5 mg/dl), hemoglobin <80 g/l, aspartate aminotransferase or alanine transaminase >3.0 × upper limit of normal, serum total bilirubin >34.2 µmol/l (2.0 mg/dl) or 3.0 × upper limit of normal, and severe renal impairment (creatinine clearance <30 ml/min or requiring dialysis).

### Treatment

Patients received pomalidomide, low-dose dexamethasone, and daratumumab in 28-day cycles until PD or unacceptable toxicity. Both pomalidomide and low-dose dexamethasone were administered orally, and daratumumab was administered intravenously. Pomalidomide 4 mg was given on days 1 to 21. Dexamethasone 40 mg (20 mg for patients >75 years of age) was given on days 1, 8, 15, and 22. Daratumumab 16 mg/kg was given on days 1, 8, 15, and 22 of cycles 1 and 2; days 1 and 15 for cycles 3 through 6; and day 1 for cycle 7 and beyond. Daratumumab dose reductions were not allowed. Thromboprophylaxis was mandatory for all patients and included low-dose aspirin, low-molecular-weight heparin, or other equivalent antithrombotic agents.

Patients experiencing grade 4 neutropenia or febrile neutropenia had their dose of pomalidomide modified. Following an event, the dose was withheld and complete blood counts were followed weekly. If the patient was not already receiving granulocyte-colony stimulating factor (G-CSF), the treating physician could initiate G-CSF at their discretion. Absolute neutrophil counts were required to be ≥500 cells/μl prior to restarting pomalidomide.

### Endpoints and assessments

The primary endpoint was ORR. Secondary endpoints were time to response (TTR), duration of response (DOR), PFS, time to progression (TTP), OS, and safety, including adverse events (AEs) and second primary malignancies (SPMs). Exploratory endpoints for cohort B included molecular, immune, and cellular markers potentially predictive of response or resistance to treatment, pharmacodynamic and mechanistic biomarkers, and health-related quality of life (HRQOL).

Tumor response was based on investigator’s assessment using local imaging review (if applicable) and central laboratory results according to modified International Myeloma Working Group criteria. Daratumumab-specific serum immunofixation electrophoresis reflex assay was performed per protocol for patients with immunoglobulin-ɣ (IgG) and -κ MM and monoclonal spike of ≤0.2 g/dl. TTR, DOR, TTP, and PFS were calculated based on the investigator’s response assessment, and all time-to-event endpoints were estimated from time of study enrollment, except DOR, which was estimated from time of response. Efficacy assessments included myeloma paraprotein, serum immunoglobulins, serum free light chain, bone marrow aspiration and/or biopsy, radiographic assessments of lytic bone lesions, and extramedullary plasmacytoma assessments.

Safety assessments included AEs, physical examination, clinical laboratory evaluations, venous thromboembolism monitoring, and pregnancy testing and counseling. AEs were coded according to Medical Dictionary for Regulatory Activities (version 20.0) and graded according to National Cancer Institute Common Terminology Criteria for Adverse Events (version 4.03). If a patient experienced the same AE multiple times, only the event of worst severity was counted. SPMs were monitored as events of interest and reported as serious AEs.

HRQOL was assessed via EuroQol EQ-5D at baseline, at day 1 of each treatment cycle prior to treatment administration, and at treatment discontinuation. In addition, the worst change from screening among all postbaseline measurements was analyzed. Overall scores were analyzed using change from baseline assessment at each postbaseline time point using a mixed model with adjustment for baseline covariates.

All patients provided bone marrow aspirate, whole blood, saliva, and normal tissue samples. Baseline and on-treatment peripheral blood samples were collected to evaluate comprehensive immune profiles, including T-cell and NK-cell subset data. All laboratory measures for safety and efficacy assessments were performed centrally, but tests that could result in dose modifications were also performed locally to facilitate treatment-related decisions during patient visits. Local laboratory results were not collected unless specifically requested by the sponsor.

### Statistical analysis

The intention-to-treat (ITT) population, defined as all enrolled patients regardless of whether they received study treatment, was used for all efficacy analyses. The efficacy evaluable (EE) population, comprising enrolled patients who received ≥1 dose of study treatment and had ≥1 postbaseline response assessment, was used to provide supportive sensitivity analyses for ORR, PFS, and OS. The safety population was defined as all enrolled patients who received ≥1 dose of study treatment. The HRQOL-evaluable population comprised patients who had baseline HRQOL evaluation and ≥1 postbaseline evaluation.

Baseline and demographic characteristics were summarized via frequency tabulations for categorical variables and descriptive statistics for continuous variables. For cohort B, a sample size of ~100 patients was deemed adequate to estimate ORR with a 95% CI of width ±9.7% about the obtained rate. Point estimates of ORR together with the 95% CI were calculated using normal approximation to the binomial distribution. Kaplan–Meier procedures were used to characterize time-to-event curves; medians and 95% CIs were estimated. Univariate summary statistics were provided for TTR.

### Study oversight

All patients provided written informed consent. This study was carried out in accordance with the principles of the Declaration of Helsinki and the International Conference on Harmonisation’s Guideline for Good Clinical Practice as well as applicable local regulations governing conduct of clinical studies. Each participating site’s institutional review board or ethics committee approved the study. This study was funded and sponsored by Bristol-Myers Squibb. The study’s sponsor compiled and maintained the data. All authors had full access to the data. This study was registered at ClinicalTrials.gov as NCT01946477.

## Results

**Fig. 1:**
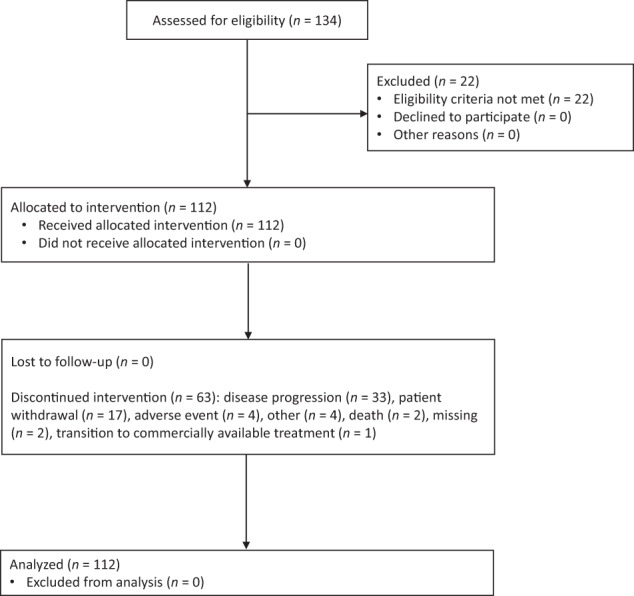
CONSORT flow diagram of cohort B of MM-014. Cohort B enrolled 112 patients.

Cohort B included 112 patients enrolled between September 2016 and December 2017 (Fig. 1). Median age was 66.5 years, and most patients (67.9%) were male (Table 1). The median time since MM diagnosis was 3.4 years. Per protocol, patients had either one (*n* = 70; 62.5%) or two prior lines of therapy (*n* = 42; 37.5%), and all patients were treated with lenalidomide in the immediate prior line of therapy; 84 patients (75.0%) were refractory to lenalidomide. The most recent prior lenalidomide dose was ≤10 mg in 54 patients (48.2%). Overall, 89 patients (79.5%) had prior exposure to a PI; 87 patients (77.7%) had received prior treatment with both lenalidomide and bortezomib. Seventy-eight patients (69.6%) had undergone prior stem cell transplant. Of 93 patients with available cytogenetic analysis, 73 patients (78.5%) were classified as standard risk, and 20 patients (21.5%) were classified as high risk (presence of del[17p], t[4;14], and/or t[14;16]).

**Table 1:** Demographic and baseline characteristics.

Characteristic	ITT population (*N* = 112)
Age, median (range), years	66.5 (39.0–83.0)
>65 years, *n* (%)	62 (55.4)
Male, *n* (%)	76 (67.9)
ECOG PS, *n* (%)	
0	44 (39.3)
1	67 (59.8)
2	1 (0.9)
Calculated R-ISS stage, *n* (%)	
I	30 (26.8)
II	53 (47.3)
III	8 (7.1)
NE	21 (18.8)
Time from MM diagnosis, median (range), years	3.4 (0.5–11.6)
Number of prior antimyeloma lines, median (range)	1 (1–2)
One prior line of therapy, *n* (%)	70 (62.5)
Two prior lines of therapy, *n* (%)	42 (37.5)
Prior therapies, *n* (%)	
LEN	112 (100)
Proteasome inhibitor^a^	89 (79.5)
BORT	87 (77.7)
CFZ	11 (9.8)
IXA	4 (3.6)
Alkylating agents	89 (79.5)
SCT	78 (69.6)
Other agents	8 (7.1)^b^
Refractory to most recent prior LEN-containing regimen, *n* (%)^c^	84 (75.0)
Duration of most recent prior LEN-containing regimen, median (range), months	23.9 (0.4–116.8)
Most recent prior LEN dose, *n* (%)	
25 mg	35 (31.3)
20 mg	4 (3.6)
15 mg	18 (16.1)
≤10 mg	54 (48.2)
Missing	1 (0.9)

*BORT* bortezomib, *CFZ* carfilzomib, *ECOG PS* Eastern Cooperative Oncology Group performance status, *ITT* intention-to-treat, *IXA* ixazomib, *LEN* lenalidomide, *MM* multiple myeloma, *NE* not evaluable, *R-ISS* revised International Staging System, *SCT* stem cell transplant.

^a^Patients may have received >1 proteasome inhibitor.

^b^Including one patient who received doxorubicin, eight patients who received etoposide, and three patients who received cisplatin. Patients may have received >1 of these agents.

^c^Refractoriness to lenalidomide was defined as being refractory to the lenalidomide-containing regimen immediately prior to study entry.

With a median follow-up of 17.2 months, 63 patients had discontinued treatment at data cutoff (February 8, 2019), and 49 patients remained on active treatment. PD was the most common cause of discontinuation (*n* = 33), followed by patient withdrawal (*n* = 17), AEs (*n* = 4), other reasons (*n* = 4), death (*n* = 2), and transition to another commercially available treatment (*n* = 1); the reason for discontinuation was missing for two patients. Median duration of treatment was 14.6 months for pomalidomide, 13.2 months for low-dose dexamethasone, and 14.4 months for daratumumab (Supplementary Table 1). Median relative dose intensity was 0.9, 0.8, and 1.0 for pomalidomide, low-dose dexamethasone, and daratumumab, respectively.

**Fig. 2:**
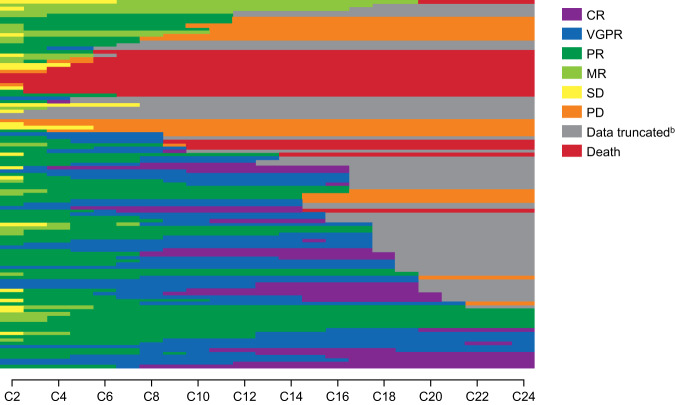
Depth of response over time in the ITT population^a^. For response missing ≤2 cycles in the range from the first available response to the last available response, the last response assessment before the gap was carried through the gap. CR complete response, ITT intention-to-treat, MR minimal response, PD progressive disease, PR partial response, SD stable disease, VGPR very good partial response. ^a^Excludes 1 patient without response. ^b^Data truncated due to discontinuation, data cutoff, or start of new treatment.

In the ITT population, 87 patients (77.7%) achieved ORR; 57 (50.9%) achieved very good partial response (VGPR) or better, and 27 patients (24.1%) achieved complete response (CR) (Table 2). In the EE population (*n* = 109), ORR was 79.8%. In both the ITT and EE populations, median TTR was 1.0 month (range, 0.8–4.8 months), and median time to best response was 3.7 months (range, 0.9–20.7 months). For most patients, depth of response increased over time (Fig. 2); 37 patients (42.5%) did not achieve their best response until ≥6 months. Median DOR was not reached in either the ITT or EE population; the 1-year DOR rate was 77.7% for both.

**Table 2:** Response (mIMWG criteria).

Response, *n* (%)	ITT population (*N* = 112)
CBR (MR or better)	96 (85.7)
ORR (PR or better)	87 (77.7)
CR	27 (24.1)
VGPR	30 (26.8)
PR	30 (26.8)
MR	9 (8.0)
SD	8 (7.1)
PD	5 (4.5)
NE	2 (1.8)
Missing	1 (0.9)

*CBR* clinical benefit response, *CR* complete response, *ITT* intention-to-treat, *mIMWG* modified International Myeloma Working Group, *MR* minimal response, *NE* not evaluable, *ORR* overall response rate, *PD* progressive disease, *PR* partial response, *SD* stable disease, *VGPR* very good partial response.

**Fig. 3:**
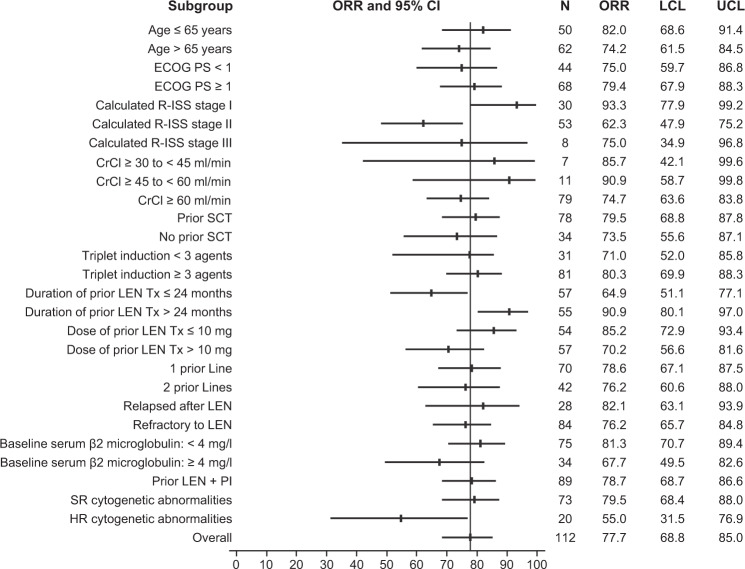
Overall response rates by subgroups. The vertical line indicates 77.7%, which was the ORR in the overall population. CrCl creatinine clearance, ECOG PS Eastern Cooperative Oncology Group performance status, HR high-risk, LCL lower control limit, LEN lenalidomide, ORR overall response rate, PI proteasome inhibitor, R-ISS Revised International Staging System, SCT stem cell transplant, SR standard risk, Tx treatment, UCL upper control limit.

In general, ORR among analyzed subgroups was consistent with that of the ITT population (Fig. 3**;** Supplementary Table 2). The ORR reported in patients with one prior line of therapy (78.6%) was similar to that observed in patients with two prior lines of therapy (76.2%). Patients who relapsed after or were refractory to lenalidomide had an ORR of 82.1% and 76.2%, respectively. In patients whose last prior dose of lenalidomide was ≤10 mg or >10 mg, 85.2% and 70.2% achieved ORR, respectively. ORR was 78.7% in patients who had prior PI and lenalidomide exposure. Patients with standard-risk vs high-risk cytogenetics had an ORR of 79.5% vs 55.0%, respectively. In addition, ORR was 90.9% in patients treated with lenalidomide for >24 months (*n* = 55) and 64.9% in patients treated with lenalidomide for ≤24 months (*n* = 57). Notably, patients with calculated Revised International Staging System stage I disease (*n* = 30) achieved an ORR of 93.3%.

**Fig. 4:**
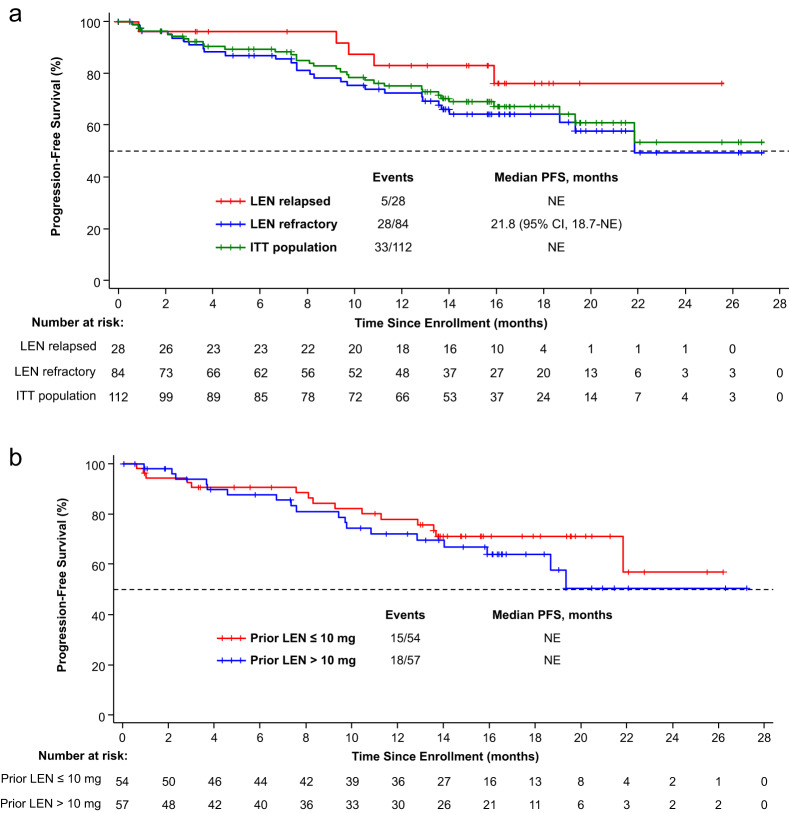
Progression-free survival. **a** Median PFS was not reached in the ITT population or in patients who relapsed after lenalidomide. Median PFS was 21.8 months in patients who were refractory to lenalidomide. **b** Median PFS was not reached in patients whose last prior dose of lenalidomide was ≤10 mg or in those whose last prior dose was >10 mg. ITT intention-to-treat, LEN lenalidomide, NE not estimable, PFS progression-free survival.

Median PFS was not reached for the ITT (Fig. 4a) or EE populations. The 1-year PFS rates were 75.1% and 75.9%, respectively. In patients who relapsed after lenalidomide, the 1-year PFS rate was 83.2%, while patients refractory to lenalidomide had a median PFS of 21.8 months and a 1-year PFS rate of 72.4%. Among patients whose last prior dose of lenalidomide was ≤10 mg, 77.9% remained alive and progression free at 1 year compared with 71.9% of those whose last prior dose of lenalidomide was >10 mg (Fig. 4b). In patients with one vs two prior lines of therapy, the 1-year PFS rate was 78.8% vs 69.0%, respectively (Supplementary Fig. 1). In patients with prior PI and lenalidomide exposure, 75.4% were alive and progression free at 1 year (Supplementary Fig. 2). The 1-year PFS rate was 82.8% vs 45.2% in patients with standard-risk vs high-risk cytogenetics, respectively (Supplementary Fig. 3). In patients whose duration of prior lenalidomide treatment was >24 vs ≤24 months, 1-year PFS rates were 85.6% vs 64.1%, respectively. Median TTP was not reached in either the ITT or EE population. OS data are not yet mature, with 20 deaths documented to date.

Measurable residual disease (MRD) assessments via next-generation sequencing (LymphoTrack, Invivoscribe, San Diego, CA) were performed in 19 patients with best response of VGPR or better and matching screen and response confirmation timepoints. Trackable clones were identified as >2.5% of all recombinant clonal sequences from the CDR3 region in 16 of 19 patients (84.2%); three patients did not have a clone sufficient for indexing. Eight of 16 patients were MRD negative (all <10^−4^ based on cellularity and lowest limit of quantification; three patients had a lower limit of quantification of 10^−5^). Twelve patients achieved CR; of these patients, seven were MRD negative at ≥6 months after study entry. Furthermore, of four patients with VGPR as best response, one patient was MRD negative. All eight MRD-negative patients and seven of eight MRD-positive patients remain in remission, with a minimum follow-up time of 16 months.

Nearly all patients (99.1%) had a treatment-emergent adverse event (TEAE). The most common nonhematologic any-grade TEAE was infections and infestations (85 patients [75.9%]), including 35 (31.3%) with upper respiratory tract infection; the most common hematologic any-grade TEAE was neutropenia (74 patients [66.1%]). Any-grade infusion-related reactions were reported in 34 patients (30.4%). The most common grade 3/4 hematologic TEAEs were neutropenia (62.5%) and anemia (17.9%), and the most common grade 3/4 nonhematologic TEAE was pneumonia (13.4%) (Table 3). Forty patients (35.7%) had ≥1 TEAE leading to reduction of pomalidomide (Table 4). The most common TEAE leading to pomalidomide dose reduction was neutropenia (20.5%). Seventy-eight patients (69.6%) had ≥1 TEAE leading to pomalidomide interruption, and 88 (78.6%) had ≥1 TEAE leading to daratumumab interruption. Pomalidomide and daratumumab dose interruptions due to TEAEs were most frequently caused by neutropenia (37.5 and 39.3%) and pneumonia (14.3 and 8.9%). Discontinuations of pomalidomide and daratumumab due to ≥1 TEAE were reported in five and eight patients (4.5% and 7.1%), respectively. Six SPMs were reported: two basal cell carcinomas, one metastatic colon cancer, one hepatic cancer, one lymphocytic leukemia, and one squamous cell carcinoma of the skin.

**Table 3:** Select grade 3/4 TEAEs.

TEAEs, *n* (%)^a^	Safety population (*N* = 112)
≥1 grade 3/4 TEAEs	104 (92.9)
Grade 3/4 hematologic TEAEs^b^	
Neutropenia	70 (62.5)
Febrile neutropenia	11 (9.8)
Anemia	20 (17.9)
Thrombocytopenia	14 (12.5)
Leukopenia	6 (5.4)
Grade 3/4 nonhematologic TEAEs^b^	
Infections and infestations	35 (31.3)
Pneumonia	15 (13.4)
Influenza	5 (4.5)
Sepsis	5 (4.5)
Parainfluenzae virus	4 (3.6)
Back pain	6 (5.4)
Insomnia	6 (5.4)
Atrial fibrillation	5 (4.5)
Dyspnea	5 (4.5)
Hyperglycemia	5 (4.5)
Hypokalemia	5 (4.5)
Hypertension	5 (4.5)
Chronic obstructive pulmonary disease	4 (3.6)
Fatigue	4 (3.6)
Hypophosphatemia	4 (3.6)

*TEAE* treatment-emergent adverse event.

^a^TEAE severity was graded according to the National Cancer Institute Common Terminology Criteria for Adverse Events version 4.03.

^b^Reported in ≥3% of the safety population.

**Table 4:** TEAEs leading to dose modifications.

TEAEs, *n* (%)	Safety population (*N* = 112)		
	Pomalidomide (*n* = 112)	Low-dose dexamethasone (*n* = 112)	Daratumumab (*n* = 112)
Patients with ≥1 TEAE leading to dose reduction	40 (35.7)	42 (37.5)	—^b^
Hematologic TEAEs^a^			
Neutropenia	23 (20.5)	2 (1.8)	—^b^
Nonhematologic TEAEs^a^			
Insomnia	0	11 (9.8)	—^b^
Hyperglycemia	0	4 (3.6)	—^b^
Peripheral edema	0	4 (3.6)	—^b^
Patients with ≥1 TEAE leading to interruption	78 (69.6)	74 (66.1)	88 (78.6)
Hematologic TEAEs^a^			
Neutropenia	42 (37.5)	38 (33.9)	44 (39.3)
Febrile neutropenia	6 (5.4)	6 (5.4)	3 (2.7)
Neutrophil count decreased	2 (1.8)	2 (1.8)	4 (3.6)
Thrombocytopenia	6 (5.4)	5 (4.5)	5 (4.5)
Leukopenia	3 (2.7)	3 (2.7)	5 (4.5)
Nonhematologic TEAEs^a^			
Infections	40 (35.7)	36 (32.1)	33 (29.5)
Pneumonia	16 (14.3)	13 (11.6)	10 (8.9)
Upper respiratory tract infection	8 (7.1)	7 (6.3)	7 (6.3)
Influenza	6 (5.4)	5 (4.5)	3 (2.7)
Bronchitis	4 (3.6)	3 (2.7)	3 (2.7)
Dyspnea	4 (3.6)	4 (3.6)	5 (4.5)
Cough	3 (2.7)	3 (2.7)	4 (3.6)
Infusion-related reaction	0	0	26 (23.2)
Patients with ≥1 TEAE leading to discontinuation^c^	5 (4.5)	8 (7.1)	8 (7.1)

*TEAE* treatment-emergent adverse event.

^a^Reported in ≥3% of patients for any drug. Patients may have had ≥1 TEAE leading to the dose modification.

^b^Per protocol, daratumumab dose reductions were not allowed.

^c^All TEAEs leading to discontinuation of each respective drug were reported in <3% of patients.

As of 15 October 2018, 108 patients were HRQOL evaluable. EQ-5D completion rates for each evaluated cycle (1–6) were ≥88%. Through all six cycles, mean changes from baseline in EQ-5D index and visual analog scale health score were stable. Minimum clinically important improvements in EQ-5D index (≥0.1) and visual analog scale health score (≥6) were achieved by 28.8% and 39.0% of patients at cycle 6, respectively. EQ-5D index values were stable, with a trend toward improvement in usual activities, pain/discomfort, and anxiety/depression.

## Discussion

The results from cohort B in MM-014 demonstrated the effectiveness and safety of pomalidomide, low-dose dexamethasone, and daratumumab in patients with RRMM immediately after failure of first- or second-line lenalidomide-based treatment. Notably, most patients (62.5%) had received only one prior line of therapy, and 75.0% were lenalidomide refractory. ORR, the primary endpoint, was 77.7%. More than half of patients achieved VGPR or better; responses deepened over time, with 42.5% of patients reaching their best response after ≥6 months of treatment, indicating the importance of long-term treatment with this regimen. Responses persisted in most patients at 1 year. Half of the patients with VGPR or better and assessable MRD achieved MRD negativity at the lowest limit of quantification. At 1 year, 75.1% of patients were alive and progression free. The reported safety profile was consistent with the known toxicities of the individual agents, and patient HRQOL was either maintained or demonstrated signs of improvement.

Pomalidomide, low-dose dexamethasone, and daratumumab benefitted most evaluated patient subgroups. ORR similar to that of the ITT population was achieved regardless of number of prior lines of therapy (one or two), refractoriness to lenalidomide, or previous bortezomib exposure. Of note, patients whose last prior dose of lenalidomide was ≤10 mg derived greater benefit from subsequent pomalidomide therapy than patients whose last prior dose was >10 mg, and patients who were treated with lenalidomide for >2 years had improved ORR vs patients who were treated for ≤2 years. More than 50% of patients with high-risk cytogenetics achieved ORR, indicating activity with this pomalidomide-based regimen in this difficult to treat population; further analyses in patients with high-risk cytogenetics are warranted. Finally, it must be noted that ORRs exceeding 90% (without the 95% confidence intervals crossing the ITT ORR of 77.7%) were observed in patients with calculated Revised International Staging System I and patients who were treated with lenalidomide for >2 years, suggesting that patients with these characteristics could derive the greatest benefit with this regimen.

Despite acquired resistance to lenalidomide in the immediate prior line of therapy, pomalidomide combined with daratumumab and dexamethasone led to deep and durable responses. Findings from the exploratory immune profile analysis demonstrated the benefits of maintaining continuous immunomodulation via sequencing pomalidomide immediately after lenalidomide, even in the context of lenalidomide resistance. The combination of pomalidomide, low-dose dexamethasone, and daratumumab did not impair the innate or adaptive immune compartments and demonstrated significant proliferative activity in CD4, CD8, and NK-cell subsets [32]. The latter finding indicates that pomalidomide-based treatment could offset daratumumab-mediated loss of NK cells [30]. Furthermore, changes from baseline, including decreases in naive T cells and selective increases in proliferative and activated T cells (with no increase in regulatory T cells), indicated a shift toward immunocompetence enhancement. Likewise, an exploratory immune profile analysis of cohort A demonstrated that pomalidomide plus low-dose dexamethasone increased CD3^+^ and CD8^+^ T-cell populations, while CD4^+^ T-cell populations remained unchanged [33]. The immune stimulation imparted by pomalidomide-based treatment in patients who relapsed after or became refractory to lenalidomide supports previous data indicating that pomalidomide is pharmacologically distinct from lenalidomide and active in the setting of lenalidomide resistance [12–20]. In addition, the higher 1-year PFS rate and ORR reported in patients who were treated with lenalidomide for >2 years vs those treated for ≤2 years, as well as the fact that responses deepened over time, may be suggestive of the benefits of uninterrupted treatment with immunomodulatory agents and tolerance to their related AEs. In clinical practice, physicians may be inclined to change class following PD on or after lenalidomide. However, these findings add to the growing body of evidence indicating that pomalidomide-based regimens, including pomalidomide with daratumumab, can overcome early-line resistance or refractoriness to lenalidomide, demonstrating that there is no evidence-based need to replace an IMiD agent with another drug class after PD on lenalidomide. Recent results with iberdomide, a novel cereblon E3 ligase modulator with enhanced tumoricidal and immunomodulatory activity, further support the beneficial role of continued immunomodulation in MM [34, 35].

Patients refractory to lenalidomide have been largely excluded from randomized trials evaluating investigational regimens against the standard control regimen of lenalidomide and dexamethasone. However, several phase 3 trials included patients refractory to lenalidomide, such as ENDEAVOR (carfilzomib plus dexamethasone; 25%), ARROW (once weekly carfilzomib plus dexamethasone; 74%), OPTIMISMM (PVd; 70%), CASTOR (daratumumab plus Vd; 28%), ELOQUENT-3 (elotuzumab, pomalidomide, and dexamethasone; 87%), and ICARIA (isatuximab, pomalidomide, and dexamethasone; 93%) [25, 36–44]. CASTOR reported ORR in the subgroup of patients refractory to lenalidomide (at last prior line of therapy); ORR was 80.5% with daratumumab plus Vd. ENDEAVOR, OPTIMISMM, and CASTOR reported median PFS values ranging from 8.6 to 9.5 months in lenalidomide-refractory patients who received the investigational treatments. These outcomes are consistent with ORR and median PFS values of the ITT populations from clinical trials that included a high percentage of lenalidomide-refractory patients (e.g., ARROW, OPTIMISMM, ELOQUENT-3, and ICARIA); among patients in the investigational arms, ORR ranged from 53.3 to 82.2%, and median PFS ranged from 10.3 to 11.5 months. While cross-trial comparisons should be interpreted cautiously, the ORR of 76.2% and 1-year PFS rate of 72.4% reported in lenalidomide-refractory patients in the current study is encouraging in the context of available data from phase 3 RRMM trials that included lenalidomide-refractory patients.

The combination of pomalidomide, low-dose dexamethasone, and daratumumab is currently approved in the United States for the treatment of patients with RRMM and ≥2 prior therapies, including lenalidomide and a PI, based on results of the phase 1b MMY1001 trial in heavily pretreated patients with RRMM (median prior lines of therapy, four) [21, 22, 31]. In contrast, patients in our trial were required to have had one or two prior lines of therapy, and 62.5% of patients had only one prior therapy. It is notable that ORR in this study was higher (77.7 vs 60.2%) and the rate of grade 3/4 neutropenia was lower (62.5 vs 76.7%). Importantly, in the current study, median PFS was not reached, and the 1-year PFS rate was 75.1%, indicating the benefit of initiating this treatment in earlier lines of therapy.

Recently published findings from the daratumumab, carfilzomib, and dexamethasone (DKd) arm of the MMY1001 study and the phase 3 CANDOR study provide valuable context regarding use of daratumumab-containing regimens in this setting [45, 46]. In MMY001, patients with RRMM (*N* = 85) and a median of two prior lines of therapy, including bortezomib and an immunomodulatory drug, received DKd [45]. Overall, 60% of patients were lenalidomide refractory. The 1-year PFS rate was 74% and ORR was 84%. Rates of grade 3/4 neutropenia, thrombocytopenia, and lymphopenia were 21%, 31%, and 24%, respectively. Twenty-four patients had cardiac TEAEs, mostly grade 3/4, which improved with carfilzomib interruption. Infusion-related reactions were common (43–60%). In CANDOR, patients with RRMM and one to three prior lines of therapy were randomized 2:1 to DKd (*n* = 312) or carfilzomib plus dexamethasone (Kd; *n* = 154) [46]. Overall, 42.3% of patients had previous exposure to lenalidomide and 33% were refractory to lenalidomide. Median PFS was not reached with DKd vs 15.8 months with Kd, and ORR was 84.3% vs 74.7% (*p* = 0.00040). Grade ≥3 cardiac failure was reported in 3.9% and 8.5% of patients receiving DKd and Kd, respectively.

Despite the higher proportion of lenalidomide-refractory patients in cohort B of MM-014, key efficacy outcomes between DKd in the MMY1001 and CANDOR trials and pomalidomide, low-dose dexamethasone, and daratumumab were similar. In addition, while the rate of grade 3/4 neutropenia in the present study was higher than in MMY1001, rates of grade 3/4 thrombocytopenia and lymphopenia were lower. Notably, infusion-related reactions also occurred less frequently in the present study (30.4%) than in MMY1001. Taken together with results from cohort B, these findings demonstrate that the pomalidomide, low-dose dexamethasone, and daratumumab regimen is an effective treatment option in the setting of lenalidomide-refractory disease. Findings from ongoing phase 3 trials evaluating pomalidomide-based regimens with monoclonal antibodies will provide further context. Interim results of the ICARIA trial evaluating pomalidomide, low-dose dexamethasone, and isatuximab in a 100% lenalidomide-exposed and 93% lenalidomide-refractory patient population (median, three prior lines of therapy) demonstrated significantly longer PFS (median, 11.5 vs 6.5 months; *p* = 0.001) and significantly higher ORR (60.4% vs 35.3%; *p* < 0.0001) compared with pomalidomide and low-dose dexamethasone [43, 44].

In clinical practice, physicians may be inclined to switch away from the immunomodulatory agent drug class after lenalidomide-based treatment failure. These findings demonstrate that pomalidomide, low-dose dexamethasone, and daratumumab is a safe and effective treatment for patients with RRMM immediately following disease progression on or after lenalidomide. These data also continue to support the integration of pomalidomide-based regimens as early as second line in the management of RRMM, even in patients refractory to lenalidomide.

## Supplementary information

Supplement

Supplemental Fig. 1. Progression-free survival by number of prior lines of therapy. Median PFS was not reached in either subgroup.

Supplemental Fig. 2. Progression-free survival in patients with prior lenalidomide and proteasome inhibitor exposure. Median PFS was not reached in this subgroup.

Supplemental Fig. 3. Progression-free survival by cytogenetic risk status. Median PFS was 10.8 months in patients with high-risk cytogenetic abnormalities and not reached in patients with standard-ris

Supplemental Material
