# Supplementary material for: Pomalidomide, dexamethasone, and daratumumab in relapsed refractory multiple myeloma after lenalidomide treatment
Source: Leukemia. 2020 May 6;34(12):3286–97. doi: 10.1038/s41375-020-0813-1 (PMC7685974; doi:10.1038/s41375-020-0813-1)
Supplement: Supplementary file 2 — Supplemental Fig. 1. Progression-free survival by number of prior lines of therapy. Median PFS was not reached in either subgroup. [file 41375_2020_813_MOESM2_ESM.pptx]

## Slide 1
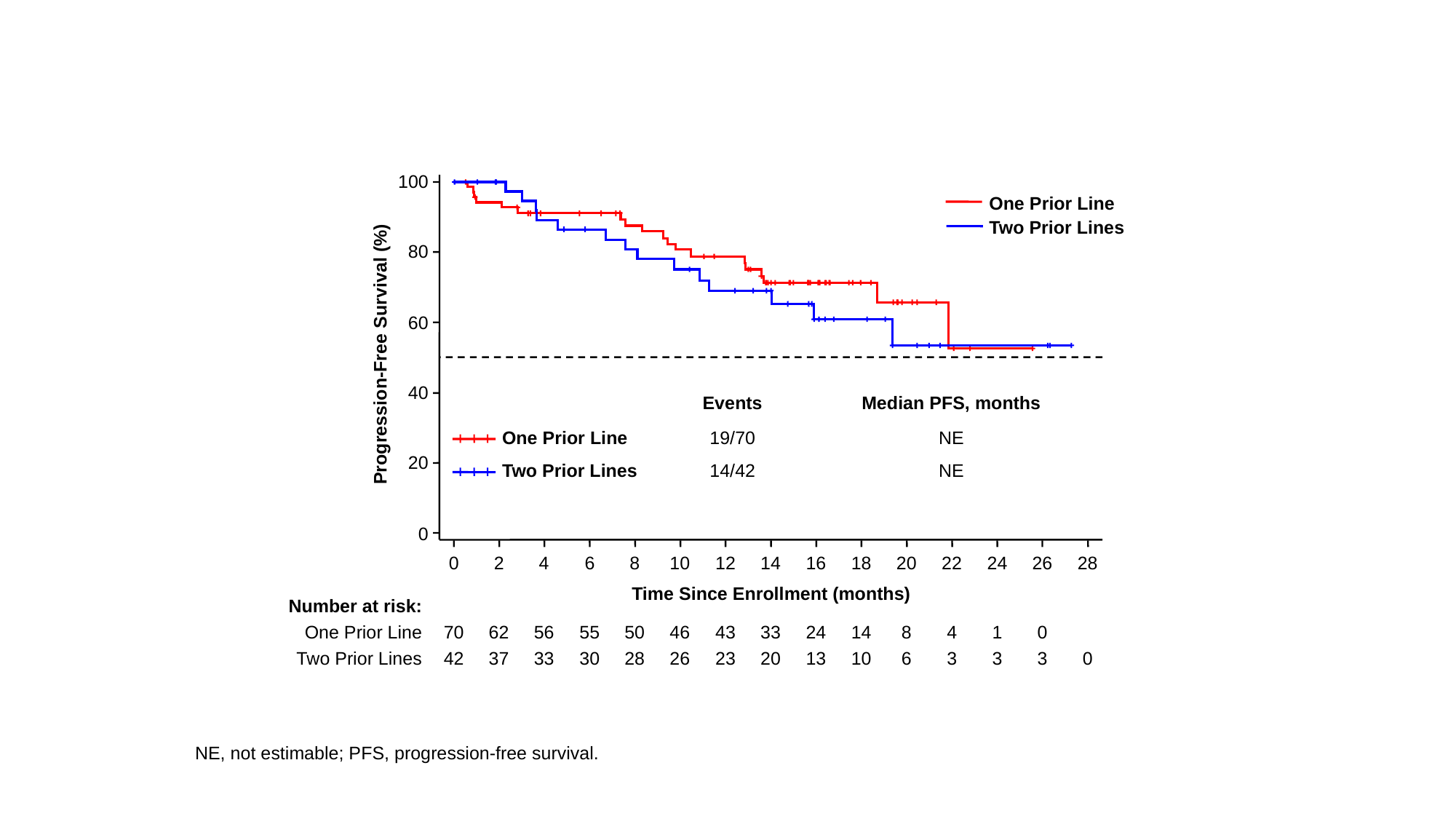

100
One Prior Line
Two Prior Lines
80
60
Progression-Free Survival (%)
40
| | Events | Median PFS, months |
| --- | --- | --- |
| One Prior Line | 19/70 | NE |
| Two Prior Lines | 14/42 | NE |
20
0
0
2
4
6
8
10
12
14
16
18
20
22
24
26
28
Time Since Enrollment (months)
Number at risk:
One Prior Line
70
62
56
55
50
46
43
33
24
14
8
4
1
0
Two Prior Lines
42
37
33
30
28
26
23
20
13
10
6
3
3
3
0
NE, not estimable; PFS, progression-free survival.
