# Supplementary material for: Pomalidomide, dexamethasone, and daratumumab in relapsed refractory multiple myeloma after lenalidomide treatment
Source: Leukemia. 2020 May 6;34(12):3286–97. doi: 10.1038/s41375-020-0813-1 (PMC7685974; doi:10.1038/s41375-020-0813-1)
Supplement: Supplementary file 3 — Supplemental Fig. 2. Progression-free survival in patients with prior lenalidomide and proteasome inhibitor exposure. Median PFS was not reached in this subgroup. [file 41375_2020_813_MOESM3_ESM.pptx]

## Slide 1
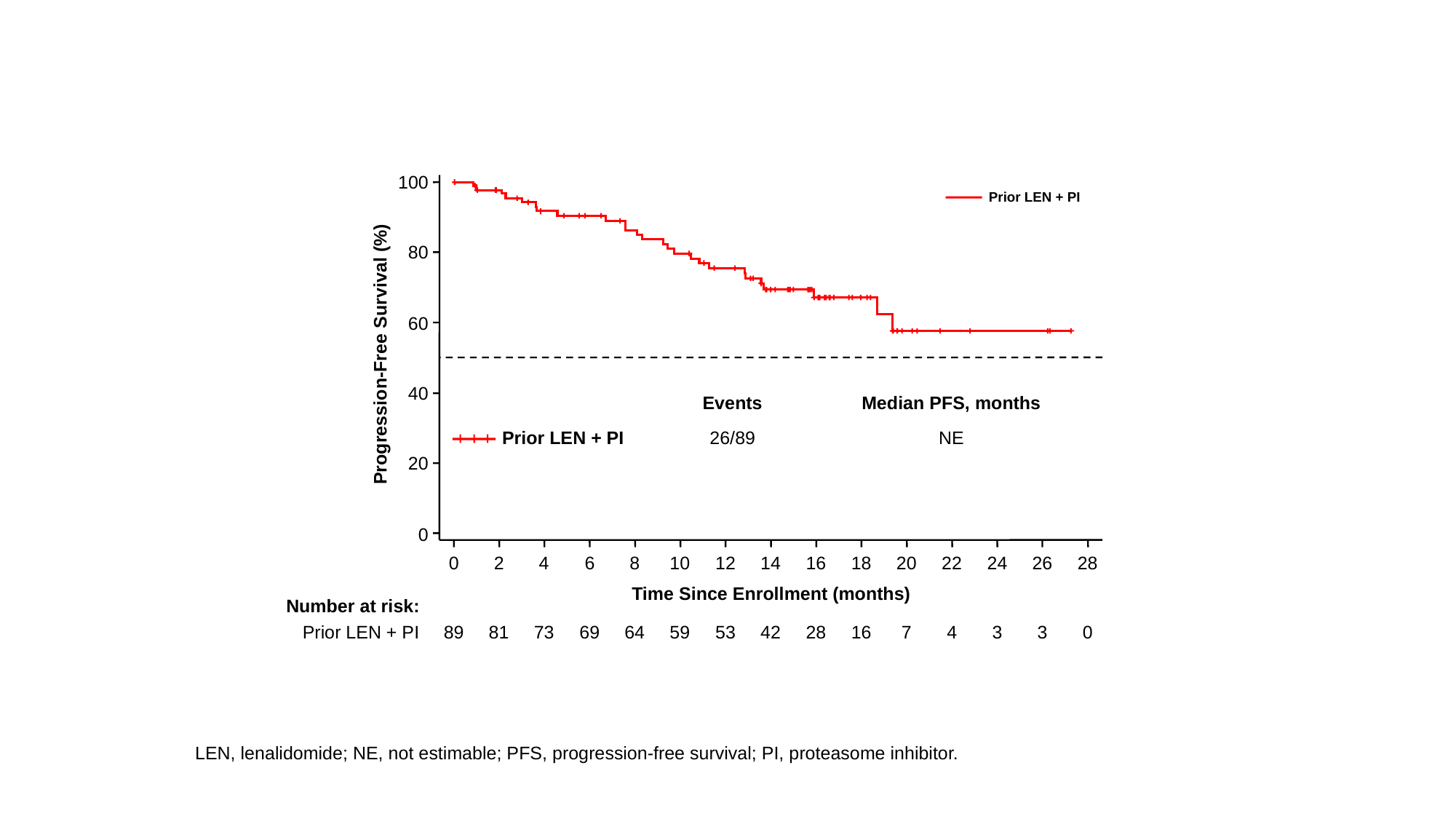

100
Prior LEN + PI
80
60
Progression-Free Survival (%)
40
| | Events | Median PFS, months |
| --- | --- | --- |
| Prior LEN + PI | 26/89 | NE |
20
0
0
2
4
6
8
10
12
14
16
18
20
22
24
26
28
Time Since Enrollment (months)
Number at risk:
Prior LEN + PI
89
81
73
69
64
59
53
42
28
16
7
4
3
3
0
LEN, lenalidomide; NE, not estimable; PFS, progression-free survival; PI, proteasome inhibitor.
