# Supplementary material for: Pomalidomide, dexamethasone, and daratumumab in relapsed refractory multiple myeloma after lenalidomide treatment
Source: Leukemia. 2020 May 6;34(12):3286–97. doi: 10.1038/s41375-020-0813-1 (PMC7685974; doi:10.1038/s41375-020-0813-1)
Supplement: Supplementary file 4 — Supplemental Fig. 3. Progression-free survival by cytogenetic risk status. Median PFS was 10.8 months in patients with high-risk cytogenetic abnormalities and not reached in patients with standard-ris [file 41375_2020_813_MOESM4_ESM.pptx]

## Slide 1
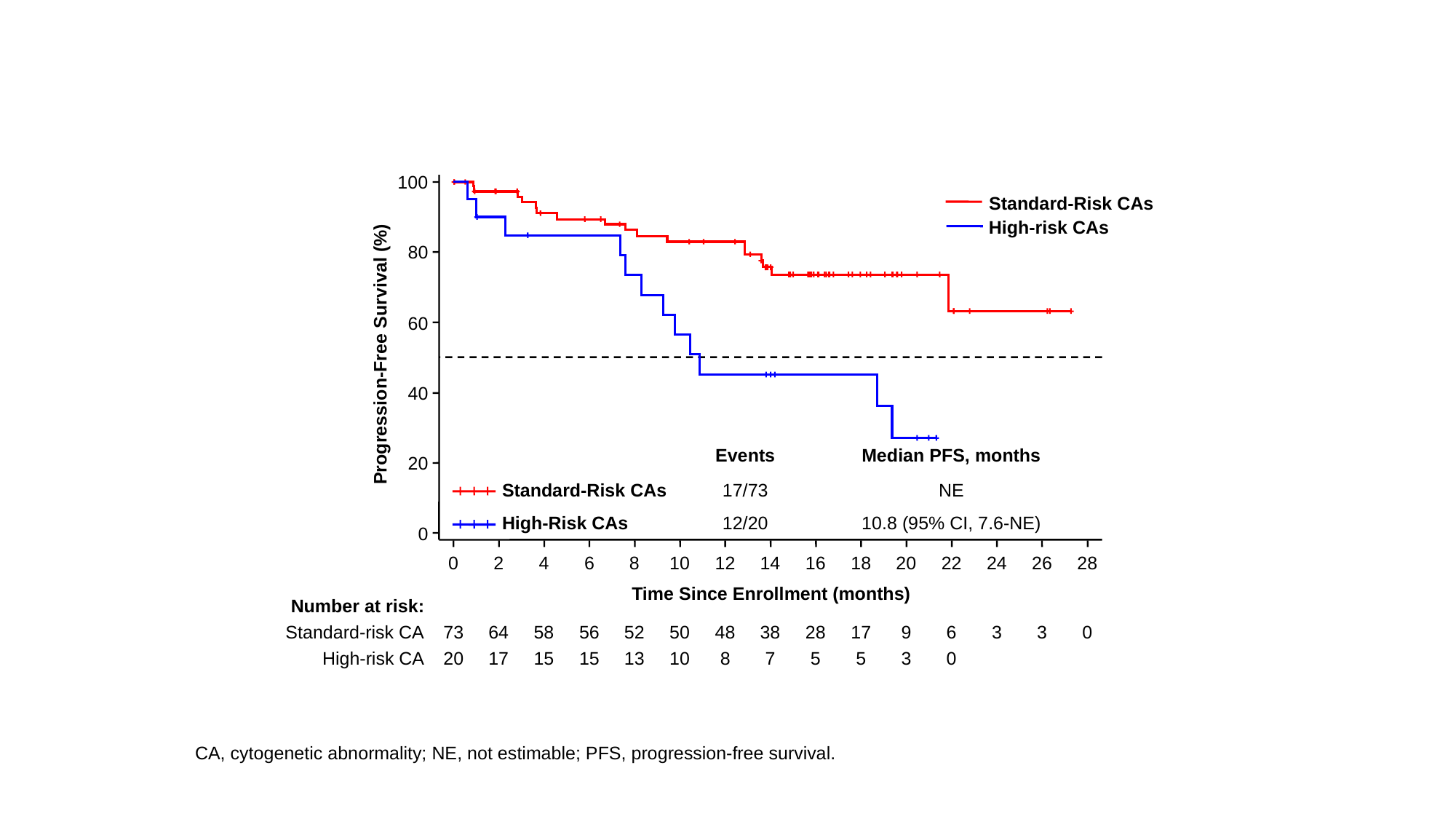

100
Standard-Risk CAs
High-risk CAs
80
60
Progression-Free Survival (%)
40
| | Events | Median PFS, months |
| --- | --- | --- |
| Standard-Risk CAs | 17/73 | NE |
| High-Risk CAs | 12/20 | 10.8 (95% CI, 7.6-NE) |
20
0
0
2
4
6
8
10
12
14
16
18
20
22
24
26
28
Time Since Enrollment (months)
Number at risk:
Standard-risk CA
73
64
58
56
52
50
48
38
28
17
9
6
3
3
0
High-risk CA
20
17
15
15
13
10
8
7
5
5
3
0
CA, cytogenetic abnormality; NE, not estimable; PFS, progression-free survival.
