# Supplementary material for: Pomalidomide, dexamethasone, and daratumumab in relapsed refractory multiple myeloma after lenalidomide treatment
Source: Leukemia. 2020 May 6;34(12):3286–97. doi: 10.1038/s41375-020-0813-1 (PMC7685974; doi:10.1038/s41375-020-0813-1)
Supplement: Supplementary file 5 — Supplemental Material [file 41375_2020_813_MOESM5_ESM.docx]

**SUPPLEMENTARY CONTENT**

**Supplemental Tables**

**Supplemental Table 1. Treatment exposure**

| **Treatment exposure** | **Safety Population (N = 112)** | | | |
| --- | --- | --- | --- | --- |
|  | **Pomalidomide**  **(n = 112)** | **Low-Dose Dexamethasone**  **(n = 112)** | **Daratumumab (n = 112)** | |
| **Treatment duration, median (range), months** | 14.6 (0.3-28.1) | 13.2 (0.3-28.3) | 14.4 (0.3-29.0) | |
| **Treatment cycles, median (range), n** | 16.0 (1.0-30.0) | 15.0 (1.0-30.0) | 15.0 (1.0-31.0) | |
| **Cumulative dose, median (range), mg** | 1079 (32.0-2443.0) | 1250 (40.0-4640.0) | 29,136.5 (56.0-73,418.0) | |
| **Relative dose intensity, median (range)** | 0.9 (0.3-1.3) | 0.8 (0.3-1.0) | 1.0 (0.0-1.3) | |
|  | | | |  |

**Supplemental Table 2. Response (mIMWG criteria) by number of prior lines of therapy, lenalidomide treatment failure status, last prior lenalidomide dose, prior proteasome inhibitor exposure, and cytogenetic risk status**

| **Response, n (%)** | **ITT Population** | | | | | | | | |
| --- | --- | --- | --- | --- | --- | --- | --- | --- | --- |
|  | **1 Prior Line of Therapy (N = 70)** | **2 Prior Lines of Therapy (N = 42)** | **LEN Relapsed**  **(n = 28)** | **LEN Refractory (n = 84)** | **Last Prior LEN Dose  ≤ 10 mg (n = 54)** | **Last Prior LEN Dose  > 10 mg**  **(n = 57)** | **Prior PI Exposure (n = 89)** | **SR CAs^a^ (n = 73)** | **HR CAs^a^ (n = 20)** |
| **CBR^b^** | 60 (85.7) | 36 (85.7) | 25 (89.3) | 71 (84.5) | 48 (88.9) | 47 (82.5) | 78 (87.6) | 63 (86.3) | 15 (75.0) |
| **ORR^c^** | 55 (78.6) | 32 (76.2) | 23 (82.1) | 64 (76.2) | 46 (85.2) | 40 (70.2) | 70 (78.7) | 58 (79.5) | 11 (55.0) |
| CR | 23 (32.9) | 4 (9.5) | 11 (39.3) | 16 (19.0) | 19 (35.2) | 7 (12.3) | 21 (23.6) | 18 (24.7) | 2 (10.0) |
| VGPR | 18 (25.7) | 12 (28.6) | 6 (21.4) | 24 (28.6) | 15 (27.8) | 15 (26.3) | 26 (29.2) | 21 (28.8) | 3 (15.0) |
| PR | 14 (20.0) | 16 (38.1) | 6 (21.4) | 24 (28.6) | 12 (22.2) | 18 (31.6) | 23 (25.8) | 19 (26.0) | 6 (30.0) |
| **MR** | 5 (7.1) | 4 (9.5) | 2 (7.1) | 7 (8.3) | 2 (3.7) | 7 (12.3) | 8 (9.0) | 5 (6.8) | 4 (20.0) |
| **SD** | 4 (5.7) | 4 (9.5) | 1 (3.6) | 7 (8.3) | 3 (5.6) | 5 (8.8) | 8 (9.0) | 5 (6.8) | 3 (15.0) |
| **PD** | 5 (7.1) | 0 | 1 (3.6) | 4 (4.8) | 3 (5.6) | 2 (3.5) | 2 (2.2) | 3 (4.1) | 2 (10.0) |
| **NE** | 1 (1.4) | 1 (2.4) | 1 (3.6) | 1 (1.2) | 0 | 2 (3.5) | 0 | 1 (1.4) | 0 |
| **Missing** | 0 | 1 (2.4) | 0 | 1 (1.2) | 0 | 1 (1.8) | 1 (1.1) | 1 (1.4) | 0 |

CA; cytogenetic abnormality; CBR, clinical benefit response; CR, complete response; HR, high-risk; ITT, intention-to-treat; LEN, lenalidomide; mIMWG, modified International Myeloma Working Group; MR, minimal response; NE, not evaluable; ORR, overall response rate; PD, progressive disease; PI, proteasome inhibitor; PR, partial response; SD, stable disease; SR, standard-risk; and VGPR, very good partial response.

^a^ HR was defined as presence of del(17p), t(4;14), and/or t(14;16). SR was defined as absence of all these abnormalities. ^b^ CBR is defined as response ≥ MR. ^c^ ORR is defined as response ≥ PR.

**Supplemental Figures**

**Supplemental Fig. 1. Progression-free survival by number of prior lines of therapy.** Median PFS was not reached in either subgroup.

**
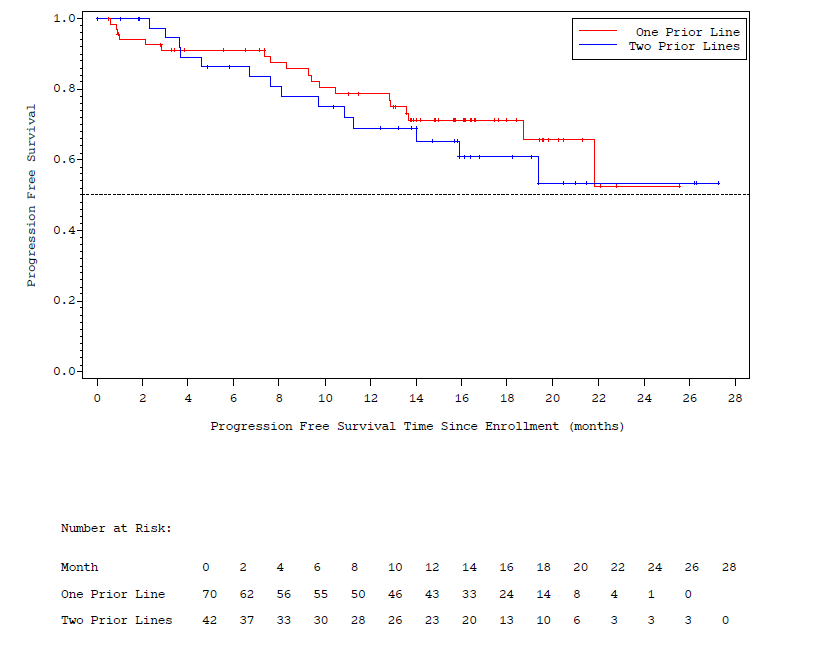
**

**Supplemental Fig. 2. Progression-free survival in patients with prior lenalidomide and proteasome inhibitor exposure.** Median PFS was not reached in this subgroup.

**
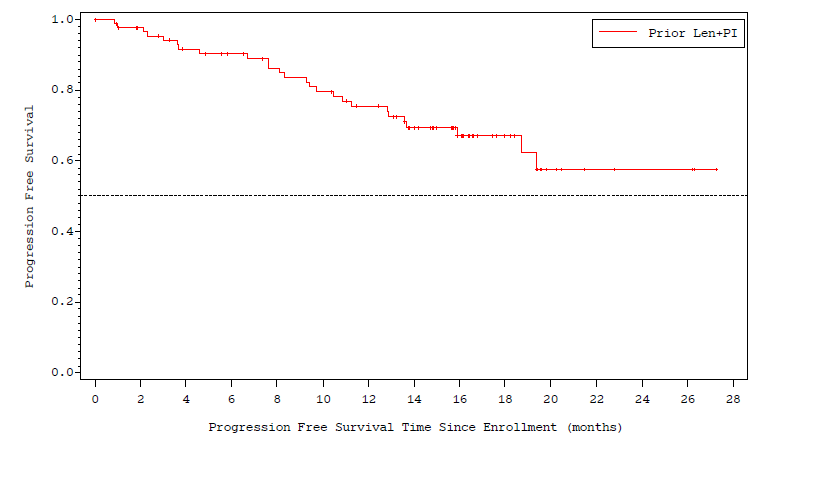
**

**Supplemental Fig. 3. Progression-free survival by cytogenetic risk status.** Median PFS was 10.8 months in patients with high-risk cytogenetic abnormalities and not reached in patients with standard-risk cytogenetics.

**
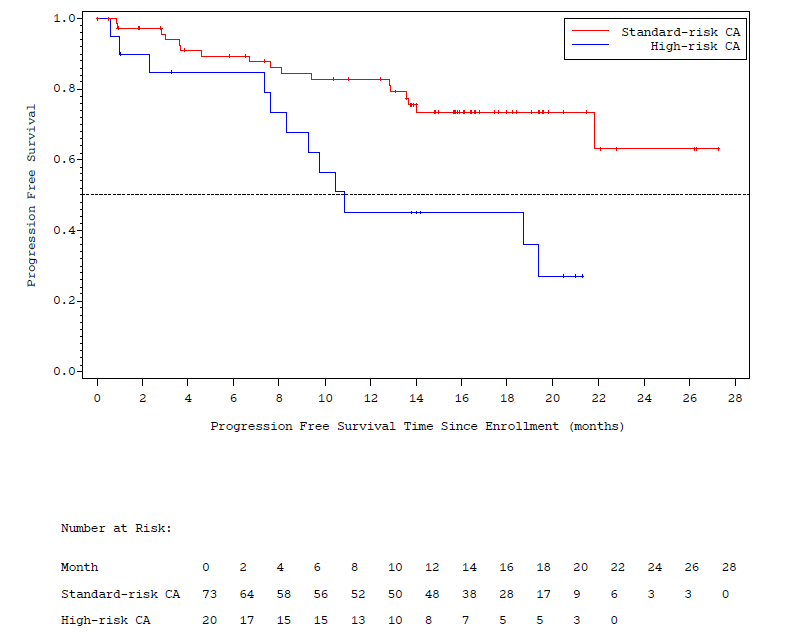
**
